# Current Status of *Campylobacter* Food Poisoning in Japan

**DOI:** 10.14252/foodsafetyfscj.D-19-00001

**Published:** 2019-08-10

**Authors:** Torrung Vetchapitak, Naoaki Misawa

**Affiliations:** 1 Graduate School of Medicine and Veterinary Medicine, University of Miyazaki, 5200 Kihara-kiyotakecho, Miyazaki 889-1692, Japan; 2 Laboratory of Veterinary Public Health, Department of Veterinary Science, Faculty of Agriculture, University of Miyazaki, 1-1 Gakuenkibanadai-nishi, Miyazaki 889-2192, Japan; 3 Center for Animal Disease Control, University of Miyazaki, 1-1 Gakuenkibanadai-nishi, Miyazaki 889-2192, Japan

**Keywords:** *Campylobacter*, food poisoning, Japan, raw chicken meat, risk profile

## Abstract

According to the annual food poisoning statistics compiled by the Ministry of Health, Labour and Welfare (MHLW) in Japan, *Campylobacter* replaced *Salmonella* and *Vibrio parahaemolyticus* as the leading bacterium responsible for food poisoning in 2003. Although in 2006 the number of cases of *Campylobacter* food poisoning was 3,439 on the basis of the MHLW statistics, it was estimated to be 1,545,363 on the basis of active surveillance, suggesting that passive surveillance yields an incidence about 450 times lower than that revealed by active surveillance. Epidemiological investigations of *Campylobacter* food poisoning in Japan have shown that chicken meat and its products are the most important sources of infection, as is the case in other industrialized nations. Over the last two decades, the consumption of fresh raw chicken meat and liver has been increasing in Japan. Although the MHLW recommends that chicken meat should only be eaten after thorough cooking, it is likely to account for much of the increased incidence of human campylobacteriosis. In response to this situation, the Expert Committee on Microorganisms/Viruses, Food Safety Commission of Japan, Cabinet Office, Government of Japan (FSCJ) has revised the previous risk profile of *C. jejuni*/*coli* in chicken meat by adding new findings for 2018. Moreover, the MHLW revised the Poultry Slaughtering Business Control and Poultry Meat Inspection Act in 2014 aiming at stepwise introduction of the Hazard Analysis Critical Control Point (HACCP) system into poultry processing plants. Subsequently, the Japanese government amended the Food Sanitation Act in 2018, requiring all food business operators to implement hygiene control based on HACCP principles as a general rule. This paper reviews the current status of *Campylobacter* food poisoning due to consumption of chicken meat in Japan and extracts the issues underlying each step of the food supply chain in order to examine the implementation of effective measures for risk management.

## 1. Introduction

The first recognized *Campylobacter* infection was reported in livestock animals in the early 20th century, when *Vibrio fetus* (now known to be *Campylobacter fetus*) was shown to be a cause of septic abortions in sheep and cattle. In 1973, Butzler et al.^1^^)^ reported the isolation of “related vibrio” from stools of healthy individuals and patients with diarrhea using a filtration method intended for isolation of *Vibrio fetus*, and the later Skirrow isolated campylobacters from patients with diarrhea using a selective medium^2^^)^. Since then, this organism has been recognized as a causal agent for human diarrhea, and currently *Campylobacter jejuni* and *C. coli* are the leading causes of enteric infections in many developed countries^3^^,^^4^^)^. In Japan, Yoshizaki and Itoh isolated *C. jejuni* from a sporadic case and from an outbreak that occurred in 1980, respectively^5^^,^^6^^)^. This led the Ministry of Health, Labour and Welfare (MHLW) in Japan to add *C. jejuni* and *C. coli* to the statistics of foodborne pathogens in 1982. Although the epidemiological data compiled by the MHLW are based on passive surveillance, the number of incidents caused by *C. jejuni*/*coli* has increased since 1997, and those incidents have remained the most commonly occurring bacterial foodborne infection since 2003, replacing *Vibrio parahaemolytics* and *Salmonella* spp. which had previously been the major bacteria responsible for food poisoning in Japan.

Epidemiological investigations of *Campylobacter* infection in humans in Japan have shown that chicken meat and its products are the most important sources of infection, as is the case for other industrialized nations. Although consumption of raw chicken meat and liver is not commonly practiced, it has been increasing in Japan during the last two decades. Although the MHLW recommends that chicken meat should only be eaten after thorough cooking, it is likely to account for much of the increased incidence of human campylobacteriosis. However, the Food Sanitation Act has not stipulated standards for edible raw chicken meat.

Against this background, the Food Safety Commission of Japan, Cabinet Office, Government of Japan (FSCJ) conducted a risk assessment of *C. jejuni*/*coli* in chicken meat in June 2009 (http://www.fsc.go.jp/hyouka/hy/hy-hyo2-campylobacter_k_n.pdf) based on the risk profile reported by the Expert Committee on Microorganisms/Viruses of the FSCJ in 2006 (http://www.fsc.go.jp/sonota/risk_profile/campylobacterjejuni.pdf). According to the risk assessment, they concluded that a combination of three countermeasures, 1) implementation of logistic slaughtering, 2) strict control of sodium hypochlorite concentration in the chiller tank, and 3) reducing the rate of raw chicken meat consumption, would be the most effective for reducing the incidence risk of *Campylobacter* food poisoning. A second proposal was to reduce contamination rate on chicken farms, rather than reducing the rate of raw chicken meat consumption. However, as the incidence of *Campylobacte*r food poisoning did not decrease after the risk assessment, due to difficulties in realistically implementing the proposals, the Expert Committee on Microorganisms/Viruses, FSCJ revised the previous risk profile of *C. jejuni*/*coli* in chicken meat in 2018 by adding new findings, specifying what is required for risk assessment, and highlighting the underlying issues remaining to be resolved (http://www.fsc.go.jp/risk_profile/index.data/180508CampylobacterRiskprofile.pdf).

In order to prevent *Campylobacter* food poisoning, it is necessary to seamlessly counteract sanitation hazards at all steps along the food chain, including farms, processing facilities, distribution, retail and kitchens. This paper reviews the current status of *Campylobacter* food poisoning due to chicken meat consumption in Japan and extracts the underlying issues at each step of the food supply chain, focusing specifically on *C. jejuni*/*coli* food poisoning.

## 2. Epidemiology of Campylobacter Food Poisoning in Japan

### 2.1 Trends in the Incidence of Campylobacter Food Poisoning

The number of cases of *Campylobacter* enteritis in Japan has been assessed independently every year in three ways: 1) reports based on the Food Sanitation Act (food poisoning statistics reported by the MHLW), 2) reports of *Campylobacter* detection mainly in outbreaks by prefectural and municipal public health institutes (PHIs) and health centers (the Infectious Agents Surveillance Report), or 3) reports of patients hospitalized due to *Campylobacter* enteritis (16 hospitals in 13 cities). To obtain an overview of food poisoning in Japan, i.e., locations, responsible foods and number of patients, the food poisoning statistics reported by the MHLW are useful.

According to the Food Sanitation Act, when medical doctors diagnose patients as having food poisoning, they are required to report each case to a nearest public health center within 24 hours after diagnosis. Then, if the public health center confirms or even suspects the case to be one of food poisoning, it is obliged to issue a prompt report and later a full report of the outbreak in detail to the MHLW via the local government.

**Fig. 1:**
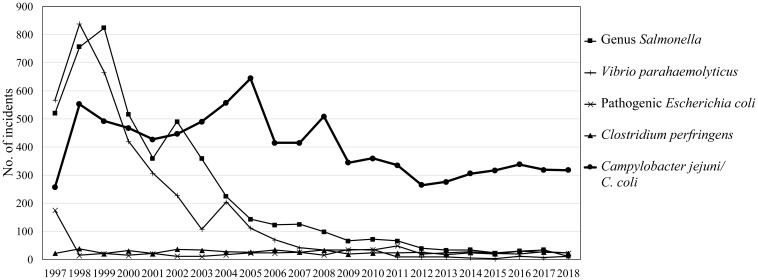
Annual changes of major bacterial foodborne diseases reported by the MHLW (1997-2018)

**Fig. 2:**
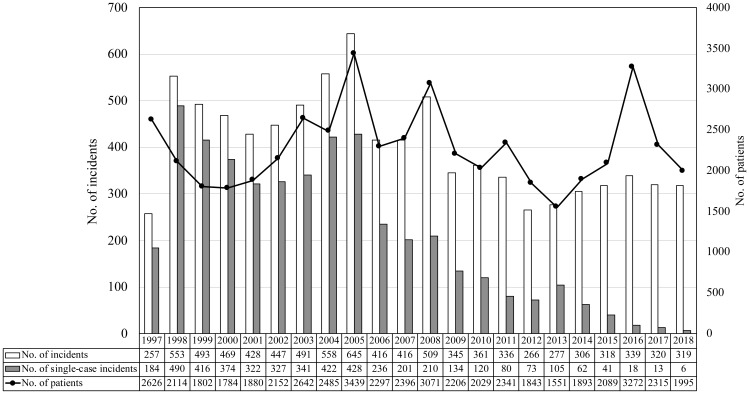
Annual changes of *Campylobacter* food poisoning in Japan (1997 -2018)

Based on the annual food poisoning statistics compiled by the MHLW, cases of *Salmonella* and *Vibrio parahaemolyticus* were most prevalent until 1997-1999, but since 2000 they have drastically decreased. On the other hand, since 2003, *Campylobacter* food poisoning has become the most common of bacterial foodborne diseases (**Fig. 1**). However, one reason for this may be the change in the MHLW food poisoning statistics in 1997, when single-case incidents began to be considered in order to grasp the overall situation, including single-cases of enterohaemorrhagic *Escherichia coli* (EHEC) food poisoning. Between 1998 and 2005, a few municipalities in Japan reported many, mostly sporadic, single-case incidents of *Campylobacter* food poisoning, and single cases accounted for 60-70% of the total. However, over the last decade, the number of single-case incidents has obviously decreased to less than 10% (**Fig. 2**). The reason for this decrease in single-case incidents since 2006 is unclear. In fact, recently, single-case incidents have hardly been reported, resulting in a low number of such incidents.

Between 1997 and 2018, the total number of incidents and cases varied from 257 to 645, and from 1,551 to 3,439, respectively (**Fig. 2**). However, there have been no reports on mortality due to campylobacteriosis since the first *Campylobacter* food poisoning statistics were reported in 1982. A previous study by Kubota et al.^7^^)^ estimated that the number of cases of *Campylobacter* food poisoning from 2005 to 2006 should have been 1,545,363 by active surveillance, although only 3,439 cases were included in the food poisoning statistics reported by the MHLW, suggesting that the incidence observed by passive surveillance is about 450 times lower than that observed by active surveillance.

### 2.2 Other Epidemiological Aspects

**Fig. 3:**
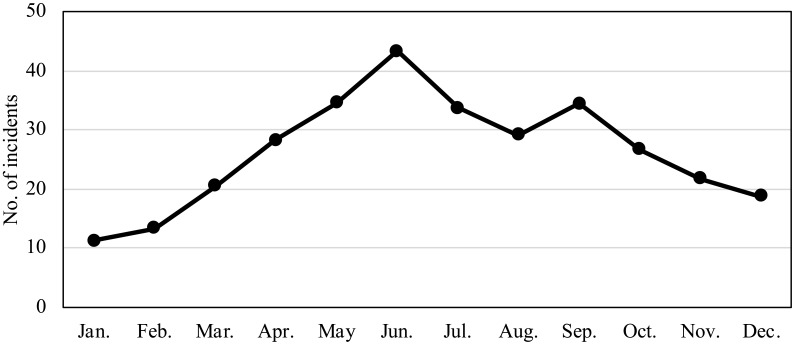
Monthly changes of *Campylobacter* food poisoning (average from 2009 to 2018)

*Campylobacter* food poisoning in Japan shows seasonal fluctuation with a major peak in the rainy season (May to July) and a minor peak in fall (September), although the incidence may still be high in the winter season (**Fig. 3**). The incidence rate is higher in males than in females, with a peak in young people aged 10 to 20 years. The rates of *C. jejuni*/*coli* isolation from adults and children in sporadic cases are around 10% and 15-25% (the highest incidence among gastrointestinal infections in children in Japan), respectively, although the rates may be underestimated as many cases are diagnosed as common cold at hospitals^8^^)^.

The *Campylobacter* Reference Center has been set up by the Reference Committee, the Associations of Public Health Laboratories for Microbiological Technology organized by the National Institute of Infectious Diseases, National Institute of Health, and 7 PHIs. The center has been collecting *C. jejuni*/*coli* isolates from patients for serotyping and drug susceptibility testing. Bacteriological examinations of the *Campylobacter* species isolated have shown that the isolation rate for *C. jejuni* was more than 90% whereas that for *C. coli* was very low. Although an outbreak of *C. fetus* associated with a barbecue was reported^9^^)^, *Campylobacter* spp. other than *C. jejuni*/*coli* have not been isolated as a foodborne pathogen due to the bacteriological protocol only for *C. jejuni*/*coli* conducted at most clinical laboratories.

According to the report on the prevalence of serotypes among isolates responsible for sporadic cases between 2009 and 2014 investigated by the 7 PHIs described above, Lior serotypes 4, 1 and 7, and Penner serogroups B (HS:2) and D (HS: 4, 13, 16, 43, 50) were dominant, respectively, though the most dominant serotypes were untyped. Moreover, resistance to antimicrobials including fluoroquinolones was reported. The percentage of isolates resistant to fluoroquinolones ranged from 35% to 50%, and the rates of quinolone resistance among human isolates gradually increased.

### 2.3 Locations of Outbreaks and Foods Responsible

**Fig. 4:**
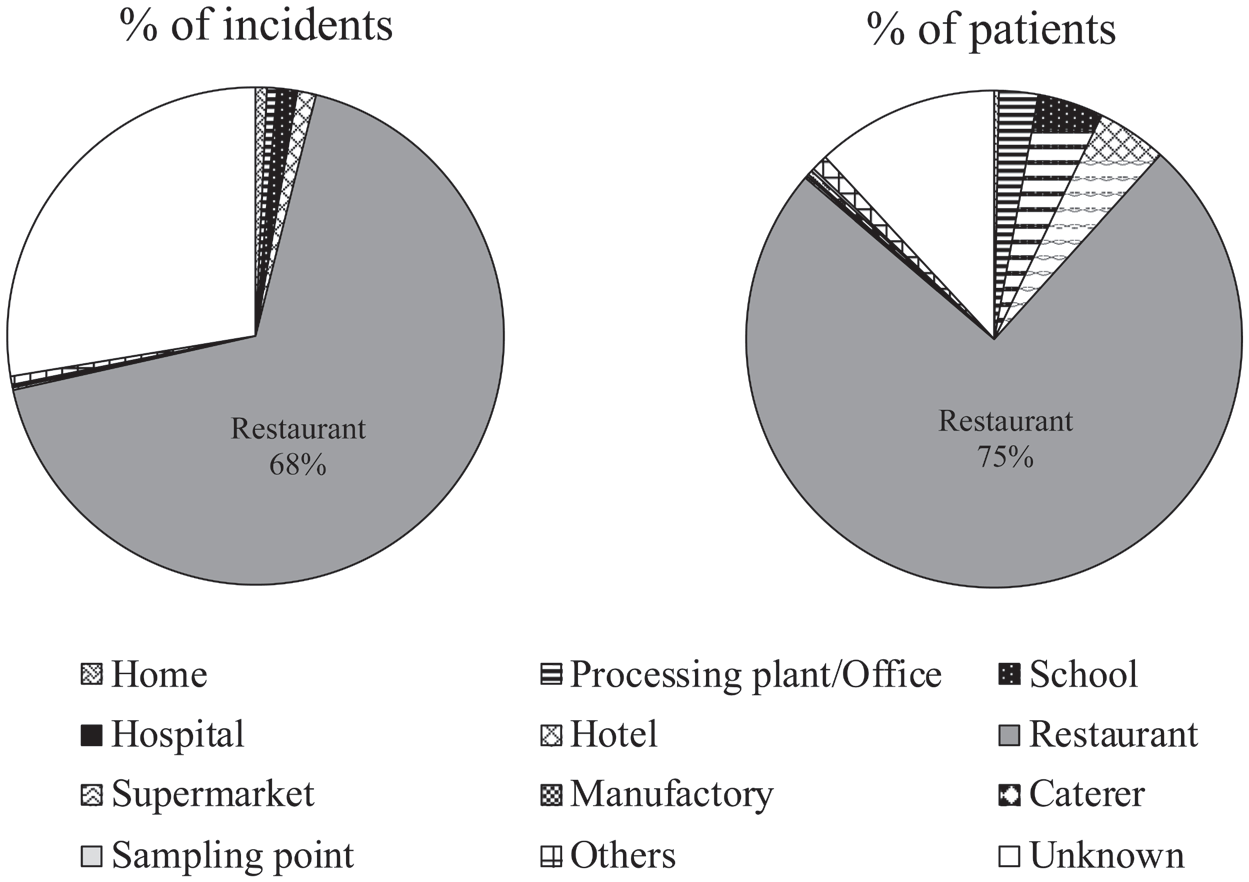
Locations of *Campylobacter* food poisoning occurred (average from 2009 to 2018)

Although the locations of sporadic incidents were unknown in many cases, outbreaks of *Campylobacter* food poisoning have commonly occurred in restaurants over the last decade, accounting for 68% and 75% of the average number of incidents and patients, respectively (**Fig. 4**). Most cases were sporadic involving a small number of patients. However, some large-scale outbreaks involving more than 100 patients due to waterborne infections and school lunches also occurred. Recently, large outbreaks involving a total of 875 patients traceable to *sushi* topped with raw or undercooked chicken meat occurred at outdoor events in Metropolitan Tokyo and Fukuoka Prefecture in 2016^10^^)^.

**Fig. 5:**
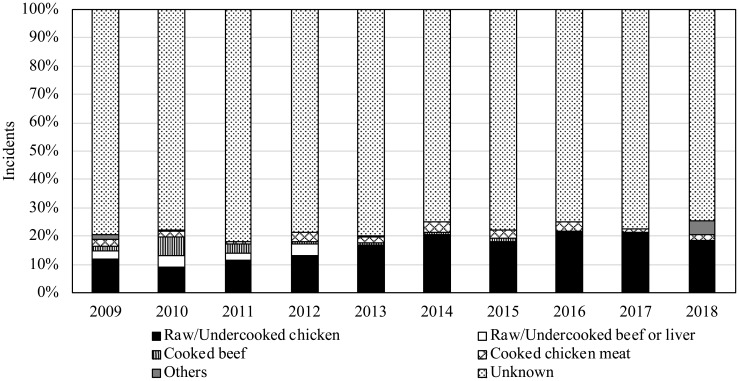
Responsible foods of *Campylobacter* food poisoning (2009-2018)

The responsible food(s) was not identified in 70-80% of the sporadic incidents, as the food(s) served was not preserved in most cases (**Fig. 5**). Furthermore, it is difficult to isolate campylobacters from food due to the small numbers of culturable bacteria present, especially in frozen foods, and/or those exposed to an aerobic environment. However, epidemiological studies have shown that fresh edible raw or undercooked chicken meat such as *torisashi*, which is sliced raw chicken meat, *tataki*, which is chicken meat burned only at the surface, and raw chicken liver and gizzard are the dominant foods responsible^3^^,^^11^^)^. Although it is unclear how long the Japanese have adopted the habit of eating fresh raw meat and inner organs, this would increase the risk of *Campylobacter* infection. Moreover, handling of poultry meat often results in infection due to secondary contamination.

**Fig. 6:**
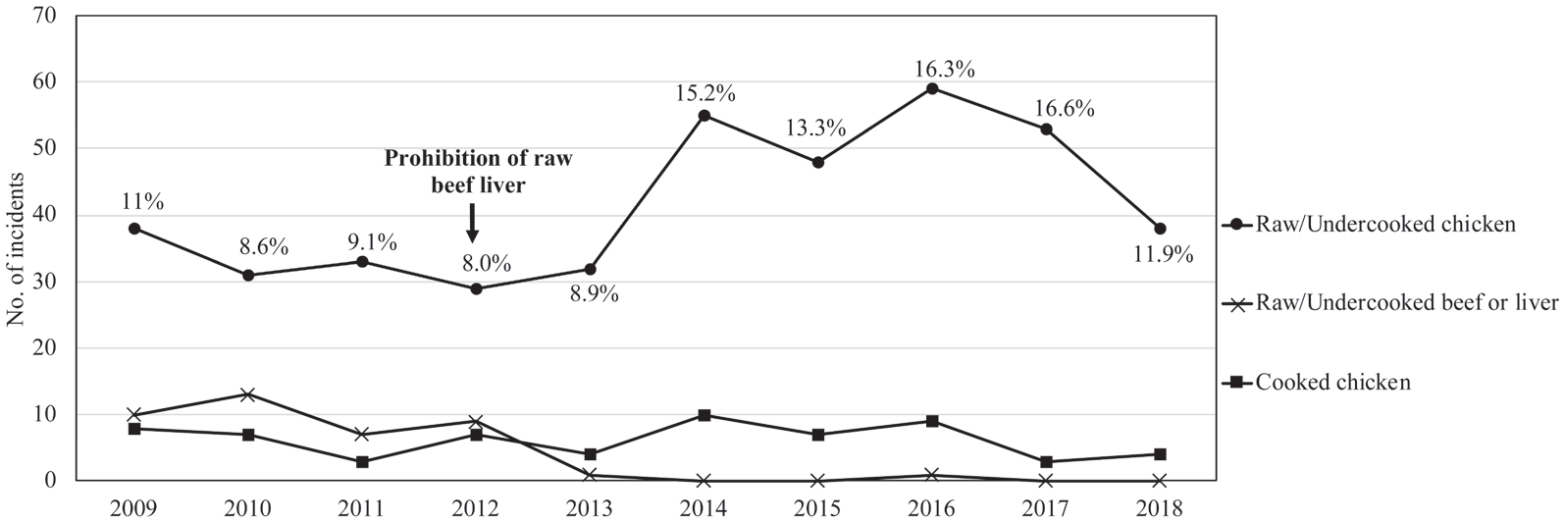
Annual changes of *Campylobacter* food poisoning caused by consumption of raw meat (2009-2018)

In 2011, a large outbreak due to consumption of raw beef products contaminated with EHEC O157:H7 occurred in several prefectures of Japan, affecting an estimated 200 patients and resulting in 5 deaths. Thereafter, the MHLW prohibited the serving of raw beef liver at restaurants due to the difficulty of decontamination of EHEC in liver, and set standards for edible raw beef, except steak, in the Food Sanitation Act of 2012 (https://www.mhlw.go.jp/topics/syokuchu/dl/110928_01.pdf). This may account for the increasing incidence of *Campylobacter* food poisoning caused by raw or undercooked chicken meat and/or liver since 2013, as the trend for eating raw or undercooked chicken products appears to have increased, thus posing a potential risk of food poisoning (**Fig. 6**). On the other hand, few milk-borne outbreaks have been reported in Japan.

## 3. Underlying Issues Preventing Reduction of Campylobacter Food Poisoning

### 3.1 Characteristics of C. jejuni/coli

*C. jejuni* usually causes diarrheal illnesses in humans. However, *Campylobacter* enteritis may be followed by septicemia^12^^)^, hepatitis^13^^)^, cholecystitis^14^^)^, hemolytic uremic syndrome (HUS)^15^^)^, meningitis^16^^)^, fetal death^17^^)^, or Guillain-Barré syndrome (GBS)^18^^)^. It is considered that the variation in clinical outcome may reflect genetic diversity among *C. jejuni* strains^19^^–^^21^^)^. Although *C. jejuni/coli* requires microaerophilic conditions (5% O_2_, 10% CO_2_ and 85% N_2_) and temperatures between 37°C and 42°C for favorable growth^2^^)^, many reports have demonstrated that *C. jejuni* possesses strategies for survival under stressful conditions^22^^–^^24^^)^.

*C. jejuni/coli* have acquired the ability to adapt to severe environmental conditions to maintain their life cycle^25^^,^^26^^)^. For example, a viable but non-culturable (VBNC) state^27^^)^, formation of biofilms^28^^,^^29^^)^, and ability for aerotolerance^30^^)^ have been reported. Although the freezing process could reduce the number of culturable organisms present on meat products^31^^,^^32^^)^, previous studies have shown that they can still survive and remain culturable for long periods in food under low temperatures and even under frozen conditions^33^^,^^34^^)^ in an ambient atmosphere. Since food industries use cold chains to keep their products fresh, this may allow *C. jejuni*/*coli* to survive in meat.

The VBNC is one possible mechanism of this pathogen to adapt environmental stress^35^^,^^36^^)^. When bacterial cells are exposed to a stressful environment, *C. jejuni* can easily enter a VBNC state accompanied by morphological changes ranging from a spiral to a coccoid form^37^^)^. Previous studies have reported that the VBNC form of *C. jejuni* can become attached to chicken skin under aerobic conditions^38^^)^ and preserve its virulence properties^39^^)^. Although the importance of the VBNC state for food poisoning remains unclear, further studies are required to clarify whether it can revert to a culturable form with restoration of virulence potential.

Several reports have shown that some strains of *C. jejuni* are aerotolerant and may survive efficiently under aerobic conditions^24^^,^^30^^,^^40^^)^, suggesting that aerotolerant strains may pose an increased risk in terms of food contamination and human infection. According to a study from Canada, approximately 36% of *C. jejuni* isolates from retail chicken meat are highly tolerant to aerobic conditions^30^^)^ and *C. jejuni* strains with increased aerotolerance survive longer on chicken meat than those sensitive to aerobic conditions^41^^)^. However, the prevalence and survival kinetics of such aerotolerance in *Campylobacter* in the food chain have never been investigated in Japan.

*C. jejuni* attaches to glass or plastic surfaces and forms a biofilm when the cells are grown without shaking *in vitro*^28^^,^^42^^)^. Biofilm formation by *C. jejuni* may be correlated with both the frequency of foodborne contamination and the consequences of infection^43^^)^. Further studies are needed to clarify the role of *C. jejuni* biofilm not only as a virulence factor, but also during survival under natural conditions or those present during food processing.

### 3.2 Primary Production Level

Healthy chickens are considered to be reservoirs for campylobacters, which colonize the intestinal tract at two to three weeks of age^44^^,^^45^^)^, suggesting that the organisms spread horizontally from the farm environment after hatching.

The authors investigated the prevalence, distribution and population dynamics of *Campylobacter* from 25 broiler flocks, comprising of 11 broiler houses and 9 farms in Kyushu, Japan, between 2013 and 2014^46^^)^. The prevalence of *Campylobacter* in broiler flocks was 44.0% (11/25), and that on farms was 88.9% (8/9). These results are similar to two previously published papers on broiler flocks in Japan, which reported a *Campylobacter* positivity rate of 43.5% between 2007 and 2008^47^^)^. Although the results are not comparable due to differences in the sampling and isolation protocols, this prevalence on Japanese broiler farms remains as high as that in other countries. As long as *Campylobacter* infections remain asymptomatic and do not affect the productivity of broilers^48^^)^, farmers do not prioritize steps to prevent such infections.

Several investigators have carried out prevalence surveillance and risk assessment of the pathogen to reduce the potential pathogen contamination risk at the farm level^47^^,^^49^^,^^50^^)^, and this has revealed that insects, wildlife, water and/or feed supply, geographical location and season may be common factors related to the higher prevalence of *Campylobacter* in broiler flocks, based on multivariate logistic regression analysis^47^^,^^49^^,^^51^^)^. Although sources of invasion responsible for *Campylobacter* contamination of broiler farms have not been identified, our epidemiological studies have demonstrated that persistent *Campylobacter* contamination mediated by an identical bacterial population in broiler houses does not occur^46^^)^.

Good biosecurity and hygiene practices are required in order to control the transmission of bacteria around farms. However, considerable differences in poultry production exist among farms in terms of farm location, chicken house structure, ventilation systems, raising systems (broiler or free-range chickens), and water and feeding systems. Therefore, it may be difficult to design and apply appropriate biosecurity procedures to each farm.

Recently, new countermeasures to control *Campylobacter* colonization in the intestinal tracts of chickens have been reported, such as probiotics^52^^)^, organic or inorganic acids as feed additives^53^^)^, bacteriocin^54^^)^, bacteriophages^55^^)^ and vacccines^56^^)^. However, these have not been firmly established as prevention tools. Therefore, it is important for farmers to estimate the extent to which chicken meat productivity can be improved by implementation of appropriate biosecurity on farms.

### 3.3 Processing Plant Level

To institutionalize poultry meat inspection systems, the MHLW passed the “Poultry Slaughtering Business Control and Poultry Meat Inspection Act” in 1990 (Ordinance of the Ministry of Health and Welfare No. 40; https://www.mhlw.go.jp/english/topics/foodsafety/dl/t-3.pdf). The poultry targeted for inspection included chickens, ducks and turkeys. At large-scale facilities processing more than 300,000 poultry annually, individual birds need to be inspected by veterinarians. On the other hand, at smaller-scale facilities authorized by the local government processing fewer than 300,000 poultry annually, inspections can be performed by licensed sanitation supervisors rather than veterinarians. Based on statistics from the MHLW, the total number of poultry processed in Japan between April 2017 and March 2018 was around 803 million (88.4% broilers, 11.4% waste chickens, 0.3% ducks and turkeys). In 2017, there were 146 large-scale and 1,776 small-scale facilities, and around 780 million broilers were slaughtered in the former. Due to such differences in scale among facilities in Japan, two styles of processing are used. In the first, evisceration is performed automatically before chilling and cutting (the so-called “nakanuki” method, which has been mostly introduced at large-scale plants, similarly to the situation in other developed countries). In the second, all muscle parts are removed from the carcass before evisceration (the so-called “sotohagi” method, which has been introduced mainly at small-scale plants).

Healthy chickens are considered to be reservoirs for campylobacters, and the organisms colonize the intestinal tract at concentrations ranging from 10^3^ to 10^9^ CFU/g intestinal content or feces^57^^–^^59^^)^. However, the degree of *C. jejuni*/*coli* colonization varies among flocks and farms^60^^)^, with positivity rates of between 0% and 100%. Once infected birds enter a processing plant, contamination of chicken carcasses with campylobacters occurs over the entire skin during defeathering and evisceration due to leakage from the crop and/or intestine. However, few quantitative data on *Campylobacter* contamination of chicken carcasses are available in Japan.

The possible causes of *Campylobacter* contamination on carcasses during poultry processing are as follows:

- Containers used for transportation of live birds are contaminated with campylobacters in fecal deposits.
- Chicken carcasses are processed together with skin.
- Expulsion and/or leakage of intestinal contents occurs during the defeathering process.
- Rupture of the intestinal tract occurs during the evisceration process.
- Contact between carcasses on the processing line.
- Low bactericidal effect of disinfectant used (sodium hypochlorite; NaOCl) to decontaminate organisms attached to the carcasses.
- Cross-contamination of chicken meat occurs easily via equipment and handling during the cutting process.

A previous study has demonstrated that microorganisms would be entrapped and retained in the feather follicles^38^^,^^61^^)^ due to morphological changes resulting from defeathering and chilling^62^^)^. However, the proportion of enlarged follicles that became closed after chilling showed no discernible relationship with the degree of *Campylobacter* contamination in different areas of the carcass skin, suggesting that campylobacters may not be confined to feather follicles as a result of the morphological changes attributable to defeathering and chilling^63^^)^.

No measures for control of pathogenic microorganisms attached to carcasses at processing plants have yet been established. To reduce contaminating bacteria including *Campylobacter* spp. on chicken carcasses in most processing plants, chilled water containing 50 to 100 ppm NaOCl is used in Japan. However, the bactericidal effect on campylobacters is not large, since organic matter often reduces the antimicrobial activity of the disinfectant^64^^)^. Therefore, the MHLW noticed the effectiveness of several sanitizers such as sodium hypochlorite, hypobromous acid water, acidified sodium chlorite (ASC), hypochlorous acid water, and peracetic acid composition for reducing the microbial contamination on the poultry carcasses at slaughter on March 2019 (https://www.mhlw.go.jp/content/11130500/000487308.pdf). However, better approaches for the control of microbial contamination throughout improved hygienic practices are still on the way of development.

According to the risk assessment guidelines for *C. jejuni*/*coli* in chicken meat reported by the FSCJ in 2009, “scheduled slaughtering” would be one of the most effective measures for reducing the risk of *Campylobacter* food poisoning for consumers. To avoid cross-contamination with *Campylobacter* during slaughter, chickens are separated into *Campylobacter*-positive and *-*negative flocks and *Campylobacter-*negative chickens are processed first^65^^–^^67^^)^. In fact, this approach is employed routinely in Iceland, Denmark and Norway. However, this slaughtering system has not become widespread in Japan due to the lack of incentive for producers and difficulties in diagnosing *Campylobacter*-positive and -negative flocks just before slaughter using a rapid on-site test, as well as processing the two groups separately on the same farm.

The Hazard Analysis Critical Control Point (HACCP) system is very effective for control of hazards during food production and processing. Japan has used this system voluntarily since 1995 for five food categories: milk and milk products, processed meat products, soft drinks, canned and retort foods, and fish products. The Japanese government then revised the Food Sanitation Act to introduce the HACCP system selectively for all food categories, instead of general hygiene management. The MHLW also revised the Poultry Slaughtering Business Control and Poultry Meat Inspection Act in 2014 to introduce the HACCP system into poultry processing plants stepwise. According to an investigation in 2015, 37 large-scale facilities (23.1%) processing 3.5 million poultry (47.1%) annually had introduced the HACCP system. In response to changes in consumer consciousness regarding food safety and globalization of foods since the last amendment of the Food Sanitation Act was promulgated in 2003, the Japanese government amended the Food Sanitation Act in 2018 to ensure that all food business operators implement hygiene control based on HACCP principles as a general rule, in addition to the prerequisite program. In line with this amendment, all poultry processing facilities in Japan must introduce the HACCP system till May 2021.

### 3.4 Retail Market and Consumer Level

In Japan, data on the levels of *Campylobacter* contamination of fresh chicken meat and internal organs (heart, liver and gizzard) in retail outlets and restaurants are limited since few reports have provided baseline data and laboratory diagnostic protocols have not been unified. Based on the available data, however, *Campylobacter* contamination of fresh chicken meat, regardless of whether it is “edible raw” or “heat cooking” meat, ranges from 40% to 80%^68^^–^^70^^)^, although data comparisons are difficult because of the various bacteriological protocols employed. According to a report on quantitative isolation of campylobacters from fresh chicken meat produced in Japan at 16 retail outlets from April 2004 to December 2011, 94 of the 154 samples (61.0%) were contaminated with *C. jejuni*/*coli* and the number of bacteria on chicken meat varied, the figures being 1.5 to 1.9 log MPN/100 g (13.6%), 2.0 to 2.9 log MPN/100 g (19.5%), 3.0 to 3.7 (16.9%) and >3.7 log MPN/100 g (9.7%)^71^^)^. These results suggested that levels of *Campylobacter* contamination varied among chicken meat examined, and that highly contaminated meat was included. Investigation of seasonal changes in contamination levels showed that the numbers of campylobacters in summer and fall were higher than in winter^72^^)^.

An investigation of bacterial contamination in retail chicken meat intended for raw consumption found that approximately 12% of samples were positive for *Campylobacter*^73^^)^. We have also investigated *Campylobacter* contamination of raw and undercooked chicken meat and raw liver labeled as “edible raw” at retail outlets and restaurants in Kyushu, Japan. The results showed that *Campylobacter* was isolated from 70% to 100% of raw chicken meat (both breast and thigh) and liver within the range 1-3 log MPN/10 g, and from 20% of undercooked meat (tataki) at 0.2 MPN/10 g (unpublished data). These data suggest that even edible raw chicken meat and liver have a risk of causing *Campylobacter* food poisoning. Therefore, it is recommended to discuss the specific hygienic control of such edible raw meat, as south area in Kyusyu have provided hygienic guideline for poultry processing.

Surprisingly, it appeared that approximately 50% of incidents of *Campylobacter* food poisoning associated with consumption of raw or undercooked chicken meat were associated with meat intended for “heat cooking”, according to data collected by the Food Sanitation Commission of the Pharmaceutical Affairs and Food Sanitation Council, MHLW, in 2018. In response to this issue, the MHLW has suggested the possibility of penalizing food business operators, who intentionally sell chicken meat for heat cooking as “edible raw meat” and are responsible for food poisoning occurred repeatedly or in a wide area, based on the “Accusation to incidents of *Campylobacter* food poisoning” (No. 5-0329 Notice of the Director of the Inspection and Safety Division, Pharmaceutical Safety and Environmental Health Bureau, MHLW, issued on March 29, 2018).

No standard currently exists for edible raw chicken meat regulated by the Food Sanitation Act. In the south Kyushu area, especially Miyazaki and Kagoshima Prefectures, edible raw or undercooked chicken meat, raw liver and gizzard are usually sold in supermarkets, suggesting that many food business operators produce edible raw and undercooked chicken meat. Therefore, the two local governments have formulated their own standards. For example, Miyazaki Prefecture has set a proposed food composition standard for edible raw and undercooked chicken meat: it should be negative for fecal coliforms, *Staphylococcus aureus*, genus *Salmonella* and genus *Campylobacter*, as determined by bacteriological testing (https://www.mhlw.go.jp/file/05-Shingikai-11121000-Iyakushokuhinkyoku-Soumuka/0000040840.pdf). Only chicken meat fulfilling all of the proposed standards can be labeled and sold as edible raw meat. However, raw liver was excluded from the standard because of the possibility of intra-organ contamination. Similarly, Kagoshima prefectural guideline was revised in 2018 (http://www.pref.kagoshima.jp/ae09/kenko-fukushi/yakuji-eisei/syokuhin/joho/documents/66345_20180614110024-1.pdf).

## 4. Future Issues to Be Resolved at Each Step of the Food Supply Chain

When the FSCJ reported the revised risk profile in 2018, they highlighted future issues to be resolved and required risk assessment based on current efforts and underlying issues as follows:

1. Current efforts to reduce the risk of *C. jejuni*/*coli* in chicken meat in Japan.
  1. Education of food business operators and consumers on the risk of eating chicken meat intended for heat cooking, edible raw, or undercooked, to achieve better understanding and spread of knowledge.
  2. Monitoring and guidance regarding proper information on labeling of chicken meat for heat cooking.
  3. Research on effective measures to reduce the risk of *Campylobacter* food poisoning at each step of the food supply.
2. Extraction of underlying issues to be resolved.
  1. Insufficient understanding of the actual contamination level due to poor quantitative surveillance.
    1. Difficulty with measures for *Campylobacter* control due to its bacteriological characteristics such as VBNC and aerotolerance status.
    2. No effect of *Campylobacter* colonization on chicken meat productivity.
    3. Differences in sampling and isolation protocols for *C*. *jejuni*/*coli*.
    4. Lack of baseline data at each step along the food chain.
    5. Lack of validation of HACCP introduction into processing facilities.
  2. *Campylobacter* food poisoning does not decrease in frequency.
    1. Chicken meat intended for heat cooking is sometimes labeled as suitable for consumption raw or undercooked.
      - Lack of proper knowledge among food business operators and consumers regarding the risk of eating chicken meat intended for heat cooking as raw or undercooked.
      - Lack of quantitative data on *Campylobacter* contamination of meat to prevent food poisoning.
      - Difficulty with implementation of scheduled slaughtering due to lack of producer incentive.
    2. Lack of effective measures for reducing the number of campylobacter colonizing the gut and contaminating the surface of chicken meat (no incentive for farmers and food business operators).
      [Farm level]
        - Chickens colonized with *Campylobacter* are asymptomatic.
        - Lack of conclusive risk management.
        - No economic benefit for farmers regardless of production of *Campylobacter*-negative chickens.
      [Processing, distribution and cooking processes]
        - Difficulty with scheduled slaughtering due to lack of rapid and simple on-site detection methods for campylobacters.
        - Cross-contamination of chicken carcasses and meat occurs easily.
        - Low awareness of secondary contamination by campylobacters during cooking.
        - In Japan chicken meat is distributed mainly fresh rather than frozen.
3. Future issues
  1. Formulation of a monitoring program.
    1. Standardization of laboratory protocol.
    2. Development of rapid and simple methods for detection of campylobacters.
    3. Continuous monitoring at each step of the food chain (farm, processing facility and distribution processes).
  2. Introduction of effective measures for risk management.
    1. Development of new approaches for risk management (validation of CCP after introduction of the HACCP system).
    2. Implementation of effective biosecurity on farms and verification of its effects.
    3. Introduction and implementation of hygiene control based on the HACCP system and verification of its effects.
    4. Spread of good practices for effective risk management.
4. Required risk assessment.
  1. Development and implementation of the monitoring plan.
    1. Clarifying the number of bacteria considered not to result in food poisoning at the consumption step.
    2. Implementation of quantitative risk assessment to set a target number of contaminating bacteria and to formulate a sampling program aimed at reducing the proportion of chicken meat that is highly contaminated with campylobacters (reference with the European Food Safety Authority (EFSA): Scientific Opinion, 2011).
  2. Introduction and implementation of effective measures for risk management.
    1. Quantitative estimation of the effect of measures for risk reduction at each step of the food supply (farm, processing and distribution steps).
      [Assumable measures for reduction of risk]
        - Do not provide edible raw chicken meat to consumers.
        - Strict regulation of the labeling and presentation for heat cooking of meat.
        - Setting of a target for reduction of the *Campylobacter* contamination level based on quantitative risk assessment.
        - Presentation of effective measures for risk management at each step of the food chain based on quantitative risk assessment.

## 5. Conclusion

Despite the increasing importance of *Campylobacter* food poisoning in Japan, effective surveillance systems for determining the health and economic burden of human campylobacteriosis, or for providing baseline data for interventions, have not been established. Since the geographical locations and climate in the northern and southern parts of Japan are quite different, the structures of broiler houses and feeding systems also differ. Moreover, large- and small-scale slaughtering facilities with different processing measures exist in Japan. Therefore, current knowledge of the most appropriate measures for controlling the different steps of the food chain is limited. Generation of such knowledge is essential to support the establishment of adequate domestic control measures.

Since the MHLW has revised the Poultry Slaughtering Business Control and Poultry Meat Inspection Act to introduce the HACCP system into poultry processing plants in Japan, it will be necessary to establish measures for validation of HACCP systems at all facilities, as is the case in the EU and USA. However, many food producers may not understand the benefits of HACCP. A questionnaire on HACCP sent to food producers who had introduced it highlighted the following major issues: improved awareness of hygiene management among workers, prompt response to customers’ claims, and emphasis on food safety of the products based on scientific evidence (http://www/mhlw.go.jp/stf/seikakunitsuite/bunya/0000104952.html). Therefore, it is important for food producers to realize the benefits of introducing the HACCP system.

Since no effective technologies for production of *Campylobacter*-free chickens and/or for decontaminating campylobacters from raw chicken meat are currently available, *Campylobacter* food poisoning should be prevented by not only avoiding consumption of edible raw meat, but also by thorough cooking and avoiding cross-contamination with other foodstuffs, particularly foods consumed without heating, via chopping boards, cooking ware or unwashed fingers. Therefore, risk communication must be provided for stakeholders at all levels of the food supply chain.
